# Evidence for Brain‐To‐Gut and Gut‐To‐Brain Pathways in Primary Care Patients With Disorders of Gut‐Brain Interaction, Inflammatory Bowel Disease and Gastroesophageal Reflux Disease

**DOI:** 10.1111/nmo.70117

**Published:** 2025-08-04

**Authors:** N. A. Koloski, M. P. Jones, A. Shah, G. Holtmann, N. J. Talley

**Affiliations:** ^1^ College of Health, Medicine and Well‐Being, University of Newcastle Callaghan New South Wales Australia; ^2^ Department of Gastroenterology & Hepatology Princess Alexandra Hospital Brisbane Queensland Australia; ^3^ University of Queensland, Faculty of Health & Behavioural Sciences St. Lucia Queensland Australia; ^4^ Department of Psychology Macquarie University Sydney New South Wales Australia; ^5^ University of Queensland Faculty of Medicine St. Lucia Queensland Australia

**Keywords:** brain/gut interaction, gastroesophageal reflux disease, general practice, inflammatory bowel disease, irritable bowel syndrome

## Abstract

**Background:**

Apart from disorders of gut‐brain interaction (DGBI), little data exist on the magnitude of the brain‐to‐gut pathway in other chronic gastrointestinal conditions such as gastroesophageal reflux disease (GERD) or inflammatory bowel disease (IBD) and what factors modify order of diagnosis. We aimed to determine the proportion of patients who received a diagnosis of a DGBI, GERD, or IBD prior to a new psychological diagnosis (gut‐to‐brain), and vice versa (brain‐to‐gut), and whether specific factors moderate the order of diagnosis.

**Method:**

Data was collected from a retrospective study of 1,129,104 patients attending general practices in the United Kingdom. Patients diagnosed with DGBI, GERD, or IBD *and* a psychological disorder (anxiety and/or depression) were included (excluding those with other organic GI disease). Information on which diagnosis appeared first was recorded. Multiple logistic regression was performed to compare a diagnosis of a DGBI, GERD, or IBD first versus a psychological diagnosis first on sociodemographic factors, medical conditions, and medication usage.

**Key Results:**

Just over half of patients were diagnosed with a psychological condition first versus after for IBS (53.9%) and ulcerative colitis (55.6%). This proportion was higher for FD (61.5%) and GERD (64.2%) but lower for Crohn's disease (45.7%). In a multivariate model, being female (OR = 1.37, 95% CI 1.25, 1.49), prior PPI (OR = 9.17, 95% CI 8.4, 10.0), antibiotic (OR = 2.54, 95% CI 2.29, 2.81) and NSAID use (OR = 1.29, 95% CI 1.18, 1.42), and prior gastroenteritis (OR = 2.19, 95% CI, 1.79, 2.67) were significant predictors for being diagnosed with GERD first. Similar results were found for DGBI.

**Conclusions & Inferences:**

Prior medication usage and gastroenteritis may play a role in generating gut‐to‐brain pathway disturbances.

## Graphical abstract

Gut–brain pathways are bidirectional, but one pathway may predominate in certain disorders. The brain‐to‐gut pathway predominates in IBS, FD, and GERD, while a gut‐to‐brain disturbance dominates in overlap IBS/FD. Prior medications and gastroenteritis may indicate gut‐brain disturbances and may lead first to DGBI or GERD and later psychological disturbance.

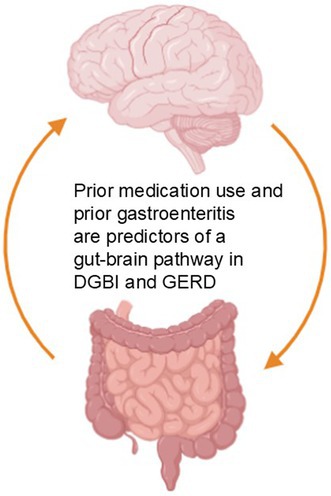

**Summary**

- The gut brain axis is bidirectional in DGBI but also in other chronic GI conditions including GERD and IBD.
- The brain to gut pathway is more dominant in GERD and functional dyspepsia, and the gut to brain pathway more dominant in Crohn's disease compared with ulcerative colitis.
- Prior medication usage of PPIs, antibiotics and NSAIDs along with a prior bout of gastroenteritis are independent predictors for being diagnosed with GERD or a DGBI first versus being diagnosed with a psychological condition first, implicating primary gut‐to‐brain pathway disturbances.

## 1 Introduction

The gut‐brain axis cross‐talk is integral to digestive health [1]. It has been hypothesized that in some chronic relapsing gastrointestinal (GI) conditions such as disorders of gut‐brain interaction (DGBI), inflammatory bowel disease (IBD) and gastroesophageal reflux disease (GERD), the gut‐brain axis direction may be disturbed [2, 3, 4]. This may explain in part the higher prevalence of psychological disorders such as anxiety and depression reported in individuals with all of these chronic GI conditions [5, 6, 7]. In the irritable bowel syndrome (IBS) and functional dyspepsia (FD) it is assumed that for some individuals, symptoms may originate first in the brain (e.g., anxiety or depression) leading to the new onset of a chronic GI symptoms (a brain‐to‐gut primary or dominant pathway) [8, 9, 10, 11]. For other individuals with IBS and FD, however, symptoms originating in the gut pre‐exist the new onset of disturbances in the brain such as anxiety (i.e., a primary gut‐to‐brain disturbance) [8, 9, 10]. Further evidence for a primary disturbance in the brain‐gut axis comes from the improvement of GI symptoms with psychological treatment in many but not all [12, 13]. On the other hand, increased duodenal microinflammation with eosinophilia in FD is associated with an increased risk of incident anxiety developing over the next decade [14].

The proportion of individuals with a DGBI who have a brain‐to‐gut versus a gut‐to‐brain disorder onset pathway is reasonably equal in population‐based studies, but a psychological disorder preceding a DGBI (i.e., brain‐gut) has been observed to be higher, with up to two thirds, in a large study of patients recruited from primary care [8, 9, 10]. Information, however, is very limited regarding the magnitude and directionality of brain‐gut pathways in important chronic organic GI diseases including inflammatory bowel disease (IBD) and gastroesophageal reflux disease (GERD).

A meta‐analysis of seven studies examining the incidence of anxiety or depression among a total of more than 150,000 patients with IBD found an increased risk of both anxiety (HR: 1.48, 95% CI: 1.29–1.70) and depression (HR: 1.55, 95% CI: 1.35–1.78) *after* a diagnosis of IBD [15]. In two studies that reported data on a brain‐gut pathway, they found among over 400,000 individuals with depression there was a 2‐fold increased risk of subsequently being diagnosed with IBD [16, 17]. While these findings support the existence of a dominant brain‐gut pathway between depression and IBD in particular, further confirmatory studies are needed. On the other hand, few studies have evaluated the temporal relationship of the brain‐gut axis in GERD. Jansson et al., in a population‐based study, found a 2.8‐fold increase in reflux risk among subjects with anxiety and depression [18]. Mendelian randomization studies support the concept that GERD is also causally linked to new onset mood disorders, but the magnitude of the risk is uncertain [19].

Little is known about any modifiable risk factors that may determine gut‐brain or brain‐gut dominance. In one of the very few studies to explore moderators of the order of incidence in DGBI, Jones et al. [10] compared brain‐gut and gut‐brain cohorts of people with a DGBI from the general population with respect to age, gender, personality, baseline anxiety, depression, a General Health Questionnaire score, and quality of life. They found baseline anxiety, depression, and neuroticism were discriminators in determining brain‐gut and gut‐brain order of incidence.

In the current study, we aimed to determine in patients with a DGBI (IBS and FD), IBD (ulcerative colitis and Crohn's disease) and GERD attending general practice, whether other variables including a range of medical conditions such as atopy and autoimmune diseases and medication usage such as antibiotics, proton pump inhibitors, nonsteroidal anti‐inflammatory drugs, and antidepressants, which have been associated with these chronic GI conditions [20, 21, 22, 23] may moderate the order of incidence of a psychological or a chronic GI diagnosis. We hypothesize that factors influencing the microbiome including exposure to antibiotics, proton pump inhibitors, and other medication usage, and a past history of gastroenteritis would be associated with developing a gut‐brain condition first.

## 2 Methods

### 2.1 Data Acquisition

The heath improvement network (THIN) provides a large database of anonymized electronic medical records collected at general practices throughout the UK [24] and has been found to be broadly representative of the demographics of the UK [24]. All recorded clinical data within THIN is coded with Read codes for medical diagnosis and drug codes for prescribed medications. Gastrointestinal disorder diagnoses from THIN have been found to be valid [25]. This procedure has been approved by the UK National Health Services' South‐East Multi‐Center Research Ethics Committee (Ref: 20/SC/0011; March 20th, 2020). Funding to obtain access to the data was provided by a grant from AusEE Inc. (Australia).

For the current study, 48,324, 57,507 and 11,265 patients were included if they had information recorded for both a psychological disorder (anxiety and/or depression) and IBS, FD, and overlap IBS/FD, respectively. In addition, to increase the reliability of a DGBI (IBS, FD, overlap IBS/FD) diagnosis, we excluded IBD, coeliac disease, peptic ulcer disease, colorectal cancer, and gastric cancer.

In terms of IBD, 677, 331, and 311 patients were included if they had information available for both a psychological disorder and IBD, ulcerative colitis, and Crohn's disease, respectively. Patients with IBD were excluded if they had a diagnosis of a DGBI (IBS and/or FD), celiac disease, peptic ulcer disease, colorectal cancer, and gastric cancer.

For the present study we also included 15,180 and 8013 patients if they had information available for a psychological disorder and GERD and overlap GERD and IBS, respectively. Patients with GERD or overlap GERD/IBS were excluded if they had a diagnosis of IBD, coeliac disease, peptic ulcer disease, colorectal cancer, and gastric cancer.

### 2.2 Data Extraction

#### 2.2.1 Determination of Order of Incidence

Information on which diagnosis (DGBI, IBD, GERD, depression or anxiety disorder) appeared first was recorded in the THIN database (January 1994 to end 2022, inclusive). Patients who had a GI condition diagnosed within 30 days of a psychological condition were excluded.

#### 2.2.2 Moderation Variables of Order of Incidence

A range of potential moderators of the order of incidence factors known to be associated with a DGBI, IBD, and GERD that were available in the THIN database were included. For medical diagnosis, these were obtained from validated read codes and included allergy, eczema, asthma, gastroenteritis, *Helicobacter pylori* diagnosis, autoimmune disease, rheumatoid arthritis, systemic lupus, and alcohol‐related diseases. We also included a diagnosis of gastroenteritis prior to a diagnosis of a chronic GI condition and psychological disorder. Medication use was obtained from drug codes and included codes for antibiotics, proton pump inhibitors (PPI), non‐steroidal anti‐inflammatory drugs (NSAIDs) and antidepressant taken prior to a diagnosis of a chronic GI condition and psychological disorder. For PPI use to be included in the analysis, PPI scripts had to be at least 30 days apart. Sociodemographic factors including age at first contact and sex were also assessed.

### 2.3 Statistical Analysis

The proportion of patients with evidence in the medical record of a psychological disorder preceding a DGBI, GERD, or IBD (brain‐gut) and vice versa is reported along with the exact 95% confidence interval (CI). Lag times between first diagnoses of gastrointestinal conditions and psychological morbidity are reported as median followed by 25th and 75th percentiles (in parentheses). Moderation of this order by the factors described previously was assessed via unconditional logistic regression in which the odds of a DGBI, GERD, or IBD diagnosis preceding a mood or anxiety disorder diagnosis was the outcome. Multiple logistic regression was performed to compare a diagnosis of a DGBI (IBS only, FD only, overlap IBS/FD), IBD (ulcerative colitis and Crohn's disease) or GERD (GERD only, overlap GERD/IBS) first versus psychological diagnosis first on a range of sociodemographic factors (age, gender), medical conditions (allergy, asthma, eczema, *Helicobacter pylori* diagnosis, autoimmune disease, rheumatoid arthritis, systemic lupus erythematosus, alcohol related diseases, gastroenteritis and prior gastroenteritis) and medication usage (antibiotics, PPIs, NSAIDs, antidepressants) prior to a diagnosis of either a DGBI, IBD, GERD, or psychological disorder. Only individual moderators that reached the criterion of *p* < 0.01 are included in the multivariate model.

## 3 Results

### 3.1 Subjects

Subjects (*n* = 1,273,605, 56.7% female, mean age at first contact = 39 years (standard deviation (SD) = 21 years)) were those individuals who had attended general practices across the United Kingdom (UK) with data available from the Health Improvement Network (THIN). The timeframe for the entire duration of the medical record for the whole sample occurred over an average period of 16 years (SD = 7 years). The prevalence of a psychological disorder in GI conditions was 41% in IBS, 33% in FD, 53% in overlap IBS/FD, 38% in Crohn's, 36% in ulcerative colitis, and 33% in GERD.

### 3.2 Order of Diagnoses

Based on the results in Table 1 we found just over half of eligible patients were diagnosed with a psychological condition first versus after for IBS (53.9%). This proportion was higher for FD (61.5%) and GERD (64.2%) with almost two thirds having a psychological diagnosis first (classified as brain‐gut). There was a slightly higher proportion of individuals with a diagnosis of overlap IBS/FD and IBS/GERD first before a psychological condition (classified as gut‐brain) (Table 1). A brain‐gut pathway (psychological condition preceding GI diagnosis) was more common in ulcerative colitis (55.6%, 95% CI 50.1, 61.0) compared with Crohn's disease (45.7%, 95% CI 40.0, 51.4; *p* = 0.02).

**TABLE 1:** Proportion of individuals with a psychological condition preceding a GI condition and vice versa.

	Total cases	Psychological condition precedes GI condition cases *N* %, 95% CI	GI condition precedes psychological condition cases *N* %, 95% CI
IBS	48,324	26,055, 53.92 (53.47, 54.6)	22,269, 46.08 (45.64, 46.53)
FD	57,507	35,367, 61.50 (61.10, 61.90)	22,140, 38.50 (38.10, 38.90)
IBS/FD Overlap	11,265	5221, 46.35 (45.42, 47.21)	6044, 53.65 (52.73, 54.58)
GERD	15,180	9747, 64.21 (63.44, 64.97)	5433, 35.79 (35.03, 36.56)
IBS/GERD Overlap	8013	3724, 46.47 (45.38, 47.57)	4289, 53.53 (52.43, 54.62)
Ulcerative Colitis	331	184 55.6 (50.1,61.0)	147 44.4 (39.0,49.9)
Crohn's Disease	311	142 45.7 (40.0,51.4)	169 54.3 (48.6,60.0)

Abbreviations: FD, functional dyspepsia; GERD, gastroesophageal reflux disease; IBS, irritable bowel syndrome.

The median lag time in years between first GI diagnosis and subsequent first psychological disorder diagnosis for IBS was 4.14 (1.79, 7.93), FD 3.73 (1.53, 7.19), overlap IBS/FD 4.16 (1.77, 7.92), Crohn's 4.79 (2.29, 8.70), ulcerative colitis 5.30 (2.48, 9.06) and GERD 4.21 (1.83, 8.27) years.

The median lag time in years between first psychological diagnosis and subsequent first GI diagnosis for IBS was 4.63 (2.04, 8.66), FD 5.57 (2.48, 9.85), overlap IBS/FD 3.91 (1.66, 7.20), Crohn's 6.24 (2.04, 11.21), ulcerative colitis 6.02 (2.27, 10.53) and GERD 6.49 (3.01, 11.13) years.

### 3.3 Moderation of Order of Diagnosis

#### 3.3.1 Irritable Bowel Syndrome

Based on univariate analysis, being female, prior PPI, antibiotic and NSAID use and prior gastroenteritis, were all significantly associated with an increased odds of being diagnosed with IBS first before a psychological disorder. Alcohol‐related diseases, *H. pylori* , asthma, allergy, and eczema were significantly associated with a reduced risk of being diagnosed with IBS versus a psychological disorder first (Table 2).

**TABLE 2:** Univariate risk factors for IBS, FD, and IBS/FD overlap preceding psychological disorder (Gut‐to‐Brain).

	IBS	FD	IBS/FD Overlap
	IBS first vs Psych first	FD First vs Psych first	IBS/FD First vs Psych first
	*N* = 22,269 *N* = 26,055	*N* = 22,140 *N* = 35,367	*N* = 6044 *N* = 5221
	*N* (%) *N* (%)	*N* (%) *N* (%)	*N* (%) *N* (%)
	OR (95% CI), *p*	OR (95% CI), *p*	OR (95% CI), *p*
Age at 1st contact	31.6 (16.0) 31.5 (14.8)	38.3 (18.1) 38.7 (15.7)	35.8 (15.8) 35.4 (14.4)
Mean age (Std dev) years	1.0 (1.0, 1.0), *p* = 0.6	1.0 (1.0, 1.0), *p* = 0.02^a^	1.0 (1.0, 1.0), *p* = 0.2
Female sex	5005 (22.5) 5174 (19.9) 1.17 (1.12, 1.22), *p* < 0.0001	10,172 (45.9) 13124 (37.1) 1.44 (1.39, 1.49), *p* < 0.0001	1511 (25.0) 1087 (20.8) 1.27 (1.16, 1.39), *p* < 0.0001
*H. pylori*	120 (0.5) 207 (0.8) 0.68 (0.54, 0.85), *p* = 0.0006	593 (2.7) 831 (2.4) 1.14 (1.03, 1.28), *p* = 0.01	177 (2.9) 158 (3.0) 0.97 (0.77, 1.21), *p* = 0.8
Allergy	3730 (16.8) 4588 (17.6) 0.94 (0.90, 0.99), *p* = 0.01	3092 (14.0) 5016 (14.2) 0.98 (0.93, 1.03), *p* = 0.5	1206 (20.0) 1127 (21.6) 0.91 (0.83, 0.99), *p* = 0.03
Rheumatoid arthritis	92 (0.4) 109 (0.4) 0.99 (0.74, 1.31), *p* = 0.9	183 (0.8) 240 (0.7) 1.22 (1.0, 1.49), *p* = 0.04	39 (0.7) 33 (0.6) 1.02 (0.64, 1.64), *p* = 0.9
Autoimmune disease	141 (0.6) 192 (0.7) 0.86 (0.69, 1.07), *p* = 0.2	112 (0.5) 224 (0.6) 0.80 (0.63, 1.01), *p* = 0.05	49 (0.8) 52 (1.0) 0.81 (0.54, 1.21), *p* = 0.3
Systemic lupus erythematosus	45 (0.2) 61 (0.2) 0.86 (0.58,1.28), *p* = 0.5	50 (0.2) 96 (0.3) 0.83 (0.59,1.18), *p* = 0.3	22 (0.4) 22 (0.4) 0.86 (0.47,1.58), *p* = 0.6
Asthma	3751 (16.8) 4669 (17.9) 0.93 (0.88, 0.97), *p* = 0.002	3335 (15.1) 5949 (16.8) 0.88 (0.84, 0.92), *p* < 0.0001	1164 (19.3) 1076 (20.6) 0.92 (0.84, 1.01), *p* = 0.07
Eczema	5889 (26.4) 7139 (27.4) 0.95 (0.91, 0.99), *p* = 0.02	5537 (25.0) 9276 (26.2) 0.94 (0.90, 0.98), *p* = 0.001	1946 (32.2) 1683 (32.2) 1.0 (0.92, 1.08), *p* = 0.9657
Alcohol‐related	256 (1.2) 420 (1.6) 0.71 (0.61, 0.83), *p* < 0.0001	514 (2.3) 1177 (3.3) 0.69 (0.62, 0.77), *p* < 0.0001	71 (1.2) 119 (2.3) 0.51 (0.38, 0.69), *p* < 0.0001
GI infection	2696 (12.1) 3227 (12.4) 0.97 (0.92, 1.03), *p* = 0.4	2433 (11.0) 3771 (10.7) 1.03 (0.98, 1.09), *p* = 0.2	970 (16.1) 940 (18.0) 0.87 (0.79, 0.96), *p* = 0.006
Prior_PPI	2095 (9.4) 1885 (7.2) 1.33 (1.25, 1.42), *p* < 0.0001	5883 (26.6) 4702 (13.3) 2.36 (2.26, 2.41), *p* < 0.0001	1072 (17.7) 530 (10.2) 1.91 (1.70, 2.14), *p* < 0.0001
Prior_antibiotic	10,942 (49.1) 12264 (47.1) 1.09 (1.05, 1.13), *p* < 0.0001	13,155 (59.4) 16312 (46.1) 1.71 (1.65, 1.77), *p* < 0.0001	2897 (47.9) 2159 (41.4) 1.31 (1.21, 1.41), *p* < 0.0001
Prior_NSAID	6795 (30.5) 7719 (29.6) 1.04 (1.0, 1.09), *p* = 0.03	9265 (41.9) 11672 (33.0) 1.46 (1.41, 1.51), *p* < 0.0001	1945 (32.2) 1468 (28.1) 1.21 (1.12, 1.32), *p* < 0.0001
Prior Antidepressant	3645 (16.4) 6094 (23.4) 0.64 (0.61, 0.67), *p* < 0.0001	4473 (20.2) 9098 (25.7) 0.73 (0.70, 0.76), *p* < 0.0001	999 (16.5) 1152 (22.1) 0.70 (0.64, 0.77), *P* < 0.0001
Prior GI infection	855 (3.8) 832 (3.2) 1.21 (1.10, 1.34), *p* = 0.0001	858 (3.9) 856 (2.4) 1.63 (1.47, 1.79), *p* < 0.0001	231 (3.8) 117 (2.2) 1.73 (1.38, 2.18), *p* < 0.0001

*Note:* Red color means the OR was significant.

^a^ The upper or lower CI boundary appears as 1.0, and these are due to rounding. Abbreviations: FD, functional dyspepsia; IBS, irritable bowel syndrome; NSAID, non‐steroidal anti‐inflammatory drugs; PPI, proton pump inhibitor.

**FIGURE 1:**
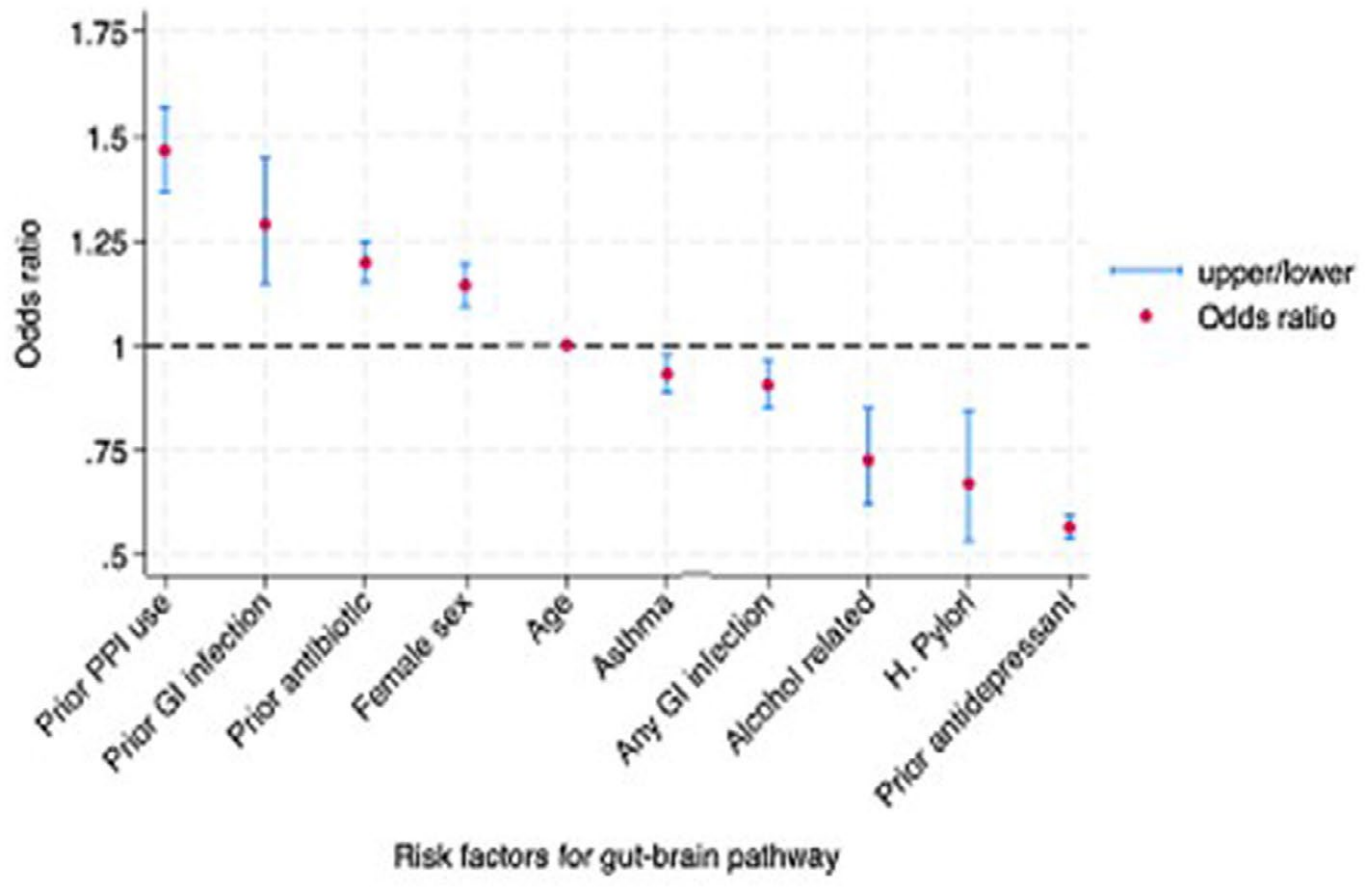
Multivariate risk factors for IBS preceding psychological disorder (Gut‐to‐Brain).

In the multivariate model, older age, being female, prior gastroenteritis, prior antibiotics, and prior PPI use remained independent predictors of being diagnosed with IBS first, whereas prior antidepressant use, alcohol‐related diseases, *H. pylori* , asthma, and gastroenteritis were independent risk factors of having a psychological disorder first (Figure 1). The area under the curve was 0.56 (95% 0.56, 0.57).

#### 3.3.2 Functional Dyspepsia

As summarized in Table 3, univariate analyses revealed that being female, prior PPI, antibiotic and NSAID use, and a prior bout of gastroenteritis, *Helicobacter pylori* and rheumatoid arthritis were significantly associated with increased odds of being diagnosed with FD first before a psychological disorder. However, asthma, prior antidepressant use, alcohol‐related diseases and eczema were significantly associated with a reduced risk of being diagnosed with FD versus a psychological disorder first (Table 2).

**TABLE 3:** Univariate risk factors for ulcerative colitis (UC) and Crohn's disease preceding psychological disorder (Gut‐to‐Brain).

	UC	Crohn's
	UC first vs Psych first	Crohn's first vs Psych first
	*N* = 147 *N* = 184	*N* = 169 *N* = 142
	*N* (%) *N* (%)	*N* (%) *N* (%)
	OR (95% CI), *p*	OR (95% CI), *p*
Age at 1st contact	43.9 (15.6) 42.4 (16.8)	35.2 (17.3) 39.9 (17.8)
Mean age (Std dev) years	1.01 (0.99, 1.02), *p* = 0.4	0.98 (0.97, 1.0), *p* = 0.02^a^
Female sex	56 (38.1) 70 (38.0) 1.0 (0.64, 1.58), *p* = 1.0	63 (37.3) 46 (32.4) 1.24 (0.77, 2.0), *p* = 0.4
*H. pylori*	1 (0.7) 4 (2.2) 0.31 (0.03, 2.92), *p* = 0.2	1 (0.6) 1 (0.7) 0.84 (0.05, 14.33), *p* = 0.9
Allergy	21 (14.3) 23 (12.5) 1.17 (0.61, 2.23), *p* = 0.6	18 (10.7) 19 (13.4) 0.77 (0.38,1.56), *p* = 0.5
Rheumatoid arthritis	1 (0.7) 3 (1.6) 0.41 (0.04, 4.21), *p* = 0.4	2 (1.2) 1 (0.7) 1.69 (0.14, 19.77), *p* = 0.7
Autoimmune disease	1 (0.7) 2 (1.1) 0.62 (0.05, 7.29), *p* = 0.7	2 (1.2) 0 (0) 1.0 (1.0, 1.0)
Systemic. lupus erythematosus	2 (1.4) 1 (0.5) 2.52 (0.22, 29.53), *p* = 0.4	2 (1.2) 0 (0) 1.0 (1.0, 1.0)
Asthma	23 (15.7) 33 (17.9) 0.85 (0.47, 1.54), *p* = 0.6	26 (15.4) 28 (19.7) 0.74 (0.41, 1.35), *p* = 0.3
Eczema	39 (26.5) 52 (28.3) 0.92 (0.56, 1.51), *p* =0 .7	44 (26.0) 39 (27.5) 0.93 (0.56, 1.55), *p* = 0.8
Alcohol‐related	5 (3.4) 5 (2.7) 1.26 (0.35, 4.56), *p* = 0.7	2 (1.2) 4 (2.8) 0.41 (0.07, 2.37), *p* = 0.3
GI infection	15 (10.2) 23 (12.5) 0.80 (0.39, 1.61), *p* = 0.5	19 (11.2) 22 (15.5) 0.69 (0.35, 1.35), *p* = 0.3
Prior PPI use	26 (17.7) 22 (12.0) 1.58 (0.85, 2.96), *p* = 0.1	38 (22.5) 20 (14.1) 1.77 (0.96, 3.25), *p* = 0.06
Prior antibiotic use	52 (35.4) 78 (42.4) 0.74 (0.47, 1.17), *p* = 0.2	66 (39.1) 60 (42.3) 0.88 (0.55, 1.39), *p* = 0.6
Prior NSAID use	42 (28.6) 53 (28.8) 0.99 (0.61, 1.61), *p* = 1.0	49 (29.0) 40 (28.2) 1.04 (0.63, 1.72), *p* = 0.9
Prior antidepressant use	23 (15.7) 44 (23.9) 0.59 (0.33, 1.04), *p* = 0.06	27 (16.0) 34 (23.9) 0.60 (0.34, 1.07), *p* = 0.08
Prior GI infection	5 (3.4) 6 (3.3) 1.04 (0.30, 3.58), *p* = 0.9	8 (4.7) 7 (4.9) 0.96 (0.33, 2.77), *p* = 0.9

^a^ The upper or lower CI boundary appears as 1.0, and these are due to rounding.

**FIGURE 2:**
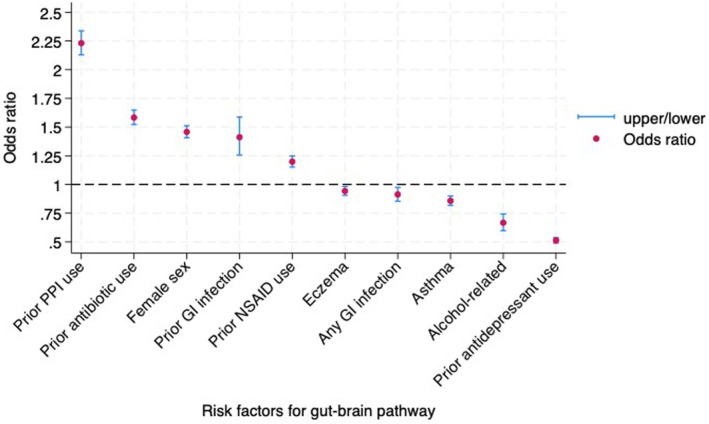
Multivariate risk factors for FD preceding psychological disorder (Gut‐to‐Brain).

In a multivariate model we found that prior PPI, antibiotic, and NSAID use, prior gastroenteritis, and being female were independent predictors of being diagnosed with FD first, whereas alcohol‐related diseases, eczema, asthma, gastroenteritis, and prior antidepressant use significantly reduced the odds of having an FD diagnosis first (Figure 2). Of clinical relevance, people diagnosed with FD first were more than twice as likely to have had prior PPI use compared with those with a psychological diagnosis first. The area under the curve was 0.64 (95% CI 0.64, 0.65).

#### 3.3.3 Overlap Irritable Bowel Syndrome and Functional Dyspepsia

Univariate analysis showed that being female, prior PPI, antibiotic and NSAID use and prior gastroenteritis, were significantly associated with an increased likelihood of being diagnosed with overlap IBS/FD first before a psychological disorder (Table 2). Alcohol‐related diseases, allergy, gastroenteritis and prior antidepressant use were significantly associated with a reduced risk of being diagnosed withoverlap IBS/FD etc. (Table 2).

**FIGURE 3:**
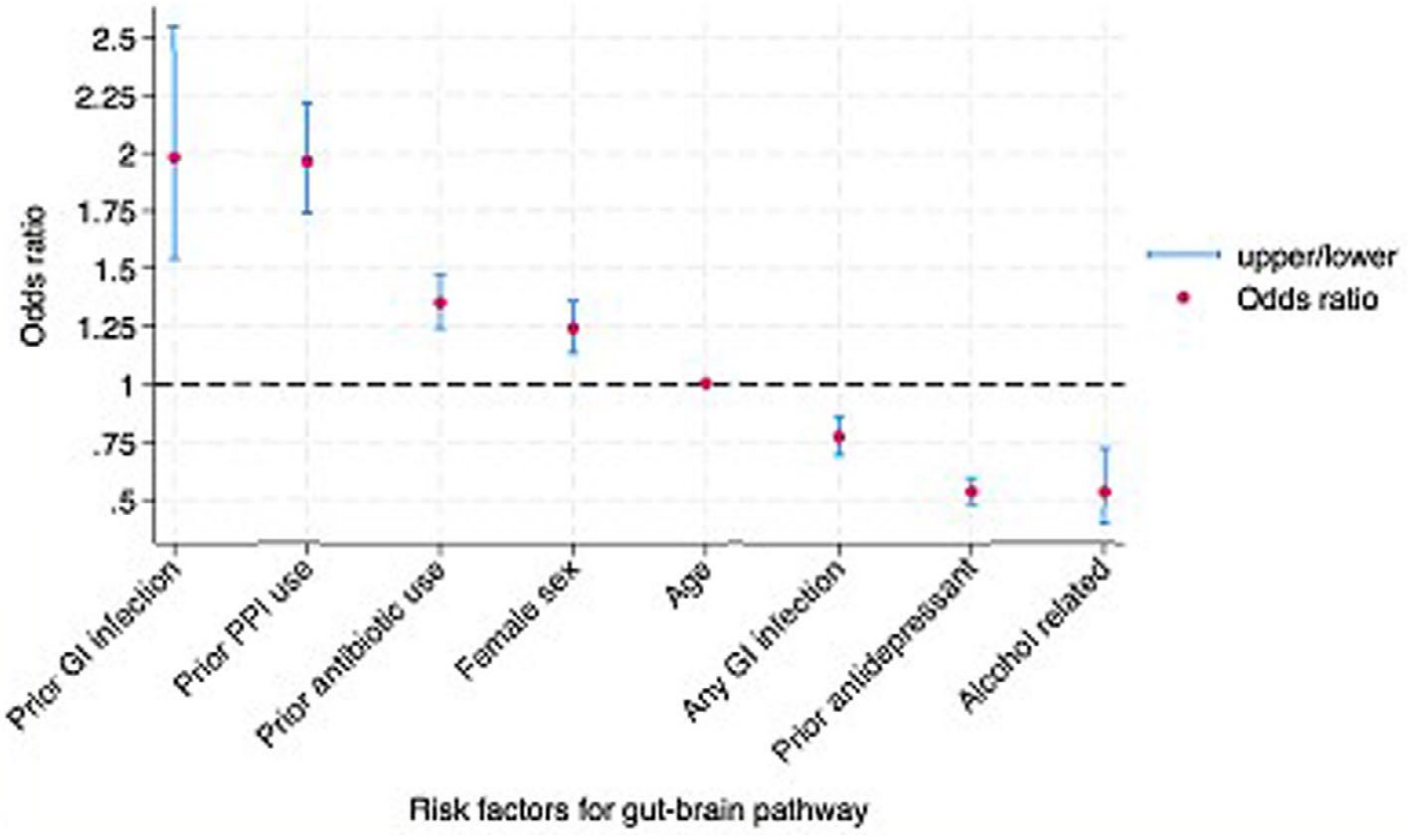
Multivariate risk factors for Overlap IBS/FD preceding psychological disorder (Gut‐to‐Brain).

Based on a multivariate model, being female, older age at first diagnosis, prior PPI and antibiotic use, and prior gastroenteritis were independent risk factors for being diagnosed with overlap IBS/FD first, whereas only alcohol‐related diseases, gastroenteritis, and prior antidepressant use remained independently associated with being diagnosed with a psychological condition first (Figure 3). The area under the curve was 0.60 (95% 0.59, 0.61).

#### 3.3.4 Inflammatory Bowel Disease

Univariately, a younger age was significantly associated with an increased odds of being diagnosed with Crohn's disease first. In a multivariate model, there were no independent risk factors associated with being diagnosed with Crohn's disease or ulcerative colitis first versus a psychological disorder.

#### 3.3.5 Gastroesophageal Reflux Disease

We found in a univariate analysis that older age at first contact, being female, prior PPI, antibiotic, NSAID and antidepressant use, and prior gastroenteritis were significantly associated with an increased odds of reporting GERD first before a psychological disorder (Table 4). Alcohol‐related diseases, autoimmune disease, and eczema were significantly associated with a reduced likelihood of being diagnosed with GERD versus a psychological disorder first (Table 4).

**TABLE 4:** Univariate risk factors for GERD and GERD/IBS Overlap preceding psychological disorder (Gut to Brain).

	GERD	GERD/IBS Overlap
	GERD first vs Psych first	GERD/IBS first vs Psych first
	*N* = 5433 *N* = 9747	*N* = 4289 *N* = 3724
	*N* (%) *N* (%)	*N* (%) *N* (%)
	OR (95% CI), *p*	OR (95% CI), *p*
Age at 1st contact	44.03 (18.9) 42.41 (15.2) 1.01 (1.00, 1.01), *p* < 0.0001 ^a^	37.32 (16.4) 36.60 (14.7) 1.0 (1.0, 1.01), *p* = 0.04 ^a^
Female sex	2066 (38.0) 2933 (30.1) 1.43 (1.33, 1.53), *p* < 0.0001	1087 (25.3) 810 (21.8) 1.22 (1.10, 1.36), *p* = 0.0002
*H. pylori*	92 (1.7) 161 (1.7) 1.03 (0.79, 1.33), *p* = 0.8	70 (1.6) 79 (2.1) 0.77 (0.55, 1.07), *p* = 0.1
Allergy	722 (13.3) 1351 (13.9) 0.95 (0.86, 1.05), *p* = 0.3	879 (20.5) 775 (20.8) 0.98 (0.88, 1.10), *p* = 0.7
Rheumatoid arthritis	45 (0.8) 60 (0.6) 1.35 (0.91, 2.0), *p* = 0.1	23 (0.5) 26 (0.7) 0.77 (0.43,1.36), *p* = 0.4
Autoimmune disease	28 (0.5) 76 (0.8) 0.66 (0.42, 1.03), *p* = 0.05	38 (0.9) 33 (0.9) 1.0 (0.62, 1.61), *p* = 1.0
Systemic lupus erythematosus	17 (0.3) 34 (0.4) 0.90 (0.49, 1.63), *p* = 0.7	16 (0.4) 6 (0.2) 2.32 (0.89, 6.05), *p* = 0.06
Asthma	1002 (18.4) 1868 (19.2) 0.95 (0.87, 1.04), *p* = 0.3	931 (21.7) 852 (22.9) 0.93 (0.84,1.04), *p* = 0.2
Eczema	1312 (24.2) 2502 (25.7) 0.92 (0.85, 1.0), *p* = 0.04	1313 (30.6) 1153 (31.0) 0.98 (0.89, 1.08), *p* = 0.7
Alcohol‐related	74 (1.4) 300 (3.1) 0.43 (0.33, 0.56), *p* < 0.0001	64 (1.5) 75 (2.0) 0.74 (0.52, 1.04), *p* = 0.07
GI infection	594 (10.9) 1006 (10.3) 1.07 (0.96,1.19), *p* = 0.2	654 (15.3) 613 (16.5) 0.91 (0.81,1.03), *p* = 0.1
Prior PPI use	4007 (73.8) 1635 (16.8) 13.94 (12.84, 15.13), *p* < 0.0001	867 (20.2) 438 (11.8) 1.90 (1.67, 2.16), *p* < 0.0001
Prior antibiotic use	4593 (84.5) 4511 (46.3) 6.35 (5.83, 6.91), *p* < 0.0001	2037 (47.5) 1552 (41.7) 1.27 (1.16, 1.39), *p* < 0.0001
Prior NSAID use	3417 (62.9) 3227 (33.1) 3.42 (3.19, 3.68), *p* < 0.0001	1342 (31.3) 1021 (27.4) 1.21 (1.09, 1.33), *p* < 0.0001
Prior antidepressant use	2608 (48.0) 2834 (29.1) 2.25 (2.10, 2.42), *p* < 0.0001	773 (18.0) 5921 (24.7) 0.67 (0.60, 0.75), *p* < 0.0001
Prior GI infection	401 (7.4) 224 (2.3) 3.39 (2.86, 4.02), *p* < 0.0001	146 (3.4) 107 (2.9) 1.19 (0.92, 1.54), *p* = 0.2

^a^ The upper or lower CI boundary appears as 1.0, and these are due to rounding.

Abbreviations: GERD, gastroesophageal reflux disease; IBS, irritable bowel syndrome.

**FIGURE 4:**
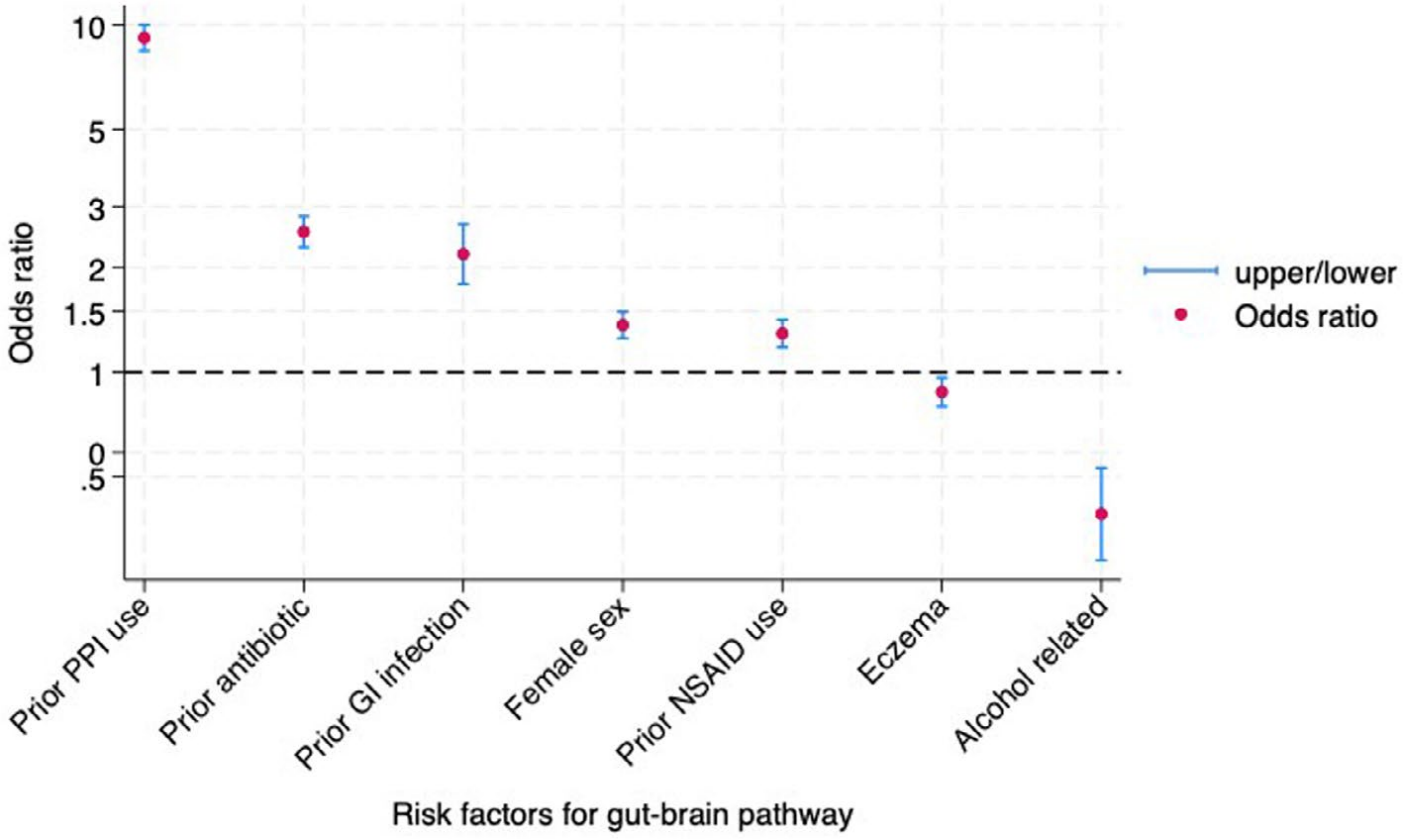
Multivariate risk factors for GERD preceding psychological disorder (Gut‐to‐Brain).

In a multivariate model, being female and prior PPI, antibiotic, and NSAID use, as well as prior gastroenteritis were independent risk factors for being diagnosed with GERD first, whereas alcohol‐related diseases and eczema were independent predictors of being diagnosed with a psychological condition first (Figure 4). Of clinical relevance, people diagnosed with GERD first were more than nine times as likely to have had prior PPI use, 2.5 times more likely to have had prior antibiotic use, and twice as likely to have prior gastroenteritis compared with those with a psychological diagnosis first (Figure 4). The area under the curve of 0.84 (95% 0.83, 0.84) suggests this model has an excellent ability to discriminate those patients diagnosed with GERD first compared with a psychological disorder first.

#### 3.3.6 Overlap GERD/IBS

Univariate analysis showed that an older age at first contact, being female, prior PPI, antibiotic, and NSAID use, but lower use of prior antidepressant use were significantly associated with an increased likelihood of reporting overlap GERD/IBS first before a psychological disorder (Table 4).

Based on a multivariate model, being female (OR = 1.89, 95% CI 1.67, 2.14, *p* < 0.001) and prior PPI use (OR = 1.20, 95% CI 1.08, 1.33, *p* = 0.001) were independent risk factors for reporting a diagnosis of overlap GERD/IBS first. The area under the curve was 0.55 (95% 0.54, 0.56).

## 4 Discussion

This study provides strong support that the brain and gut interaction is bidirectional in chronic GI disorders including DGBI, GERD, and IBD. Further, we found that prior medication use and gastroenteritis are potential moderators of a chronic GI diagnosis preceding a psychological disorder.

Despite confirming previous reports that a higher proportion of patients from primary care with a DGBI (IBS and FD) experience a psychological condition before their DGBI condition (a brain‐to‐gut pathway), we observed lower rates of a psychological condition preceding a DGBI diagnosis first for IBS only (54%) and FD only (62%), compared to prior UK data (78% and 73%, respectively) [10]. This difference may be due to our exclusion of organic conditions and use of a stricter definition for functional dyspepsia. Notably, this is the first study to quantify the magnitude of order of incidence for overlap IBS/FD, with our data suggesting that slightly more than half of patients (53.7%) received a diagnosis of overlap IBS/FD occurring first before a psychological disorder was diagnosed, suggesting a gut‐to‐brain pathway may be only slightly more common in patients with an overlapping DGBI. This may relate to a more severe phenotype in overlap IBS/FD [26] and possibly greater low‐grade intestinal inflammation (e.g., mast cells, eosinophils) may be more extensive in overlap IBS/FD since both the upper and lower parts of the GI tract are involved [27] or systemic inflammation (e.g., cytokine release) as a result of an infection, dietary antigens, or microbiota alteration may be higher in overlap IBS‐FD [27, 28, 29]. Similarly, overlap IBS/FD has also been associated with greater levels of psychological distress [30] (and release of stress hormones) which may explain why the brain‐gut pathway may also be activated in a significant proportion of patients with overlap IBS/FD.

Interestingly, up to two thirds of patients with GERD had a diagnosis of a psychological condition preceding their GERD diagnosis, suggesting the high but under‐estimated importance of brain‐to‐gut cross talk in this disease. A population‐based study [18] did find a 2.8‐fold increase in reflux risk among subjects with anxiety and depression, but this study relied on retrospective recall. Mendelian randomization studies when pooled support the concept that GERD can cause new onset mood disorders, while anxiety or depression can also cause new onset GERD [19]. It is unclear why a psychological diagnosis might precede GERD, but stress can significantly impact the upper GI tract, causing increased visceral sensitivity [31] and mast cells in the esophagus, as seen in non‐erosive reflux disease [32]. Emerging evidence also links duodenal microinflammation with increased eosinophils (also observed in functional dyspepsia) to an increased risk of new onset GERD [33], possibly due to exaggerated duodeno‐gastro‐esophageal reflexes that alter gastric motility and increase transient lower esophageal sphincter relaxations [34].

With respect to IBD, this study extends previous findings that patients with depression are at a twofold risk of developing IBD [16, 17] by also including anxiety as part of a psychological disorder assessment. Although a gut‐to‐brain pathway was significantly more common in Crohn's disease (54%) compared with ulcerative colitis (44%) this difference is within random sampling variation of equal proportions and is based on a smaller sample than the DGBI findings. Hence, we would interpret these results as being consistent with equal rates of GI and psychological morbidity coming first in IBD. Nevertheless, there is evidence that the prevalence of anxiety and depression in cross‐sectional studies is higher in Crohn's disease versus ulcerative colitis patients [7]. The bidirectional nature of the brain‐gut axis in IBD is further supported by animal models of colitis, where it has been observed that psychological disorders can lead to gut inflammation, while other studies have shown that systemic inflammation may directly contribute to psychological distress [35, 36, 37]. Microbiome alterations may also be important in explaining psychological problems in IBD by altering levels of tryptophan, a neurotransmitter associated with depression and anxiety [38]. Our results overall are consistent with Mendelian randomized studies that reported depression was causally linked to developing new‐onset ulcerative colitis [39] and Crohn's disease [40].

There are almost no data available on potential risk factors that may moderate the order of incidence of a chronic GI condition (DGBI, GERD, IBD) or psychological diagnosis. Being female was independently associated with a gut‐to‐brain pathway in all chronic GI conditions and diseases under consideration except IBD. We found strong support for prior PPI use as an independent predictor for a gut‐to‐brain pathway with all the chronic GI conditions under investigation. We required patients to have been prescribed PPIs at least 30 days apart to eliminate once off short‐term use of PPIs as well as prior to a diagnosis of a GI or psychological disorder. PPI use can impact the small intestinal microbiome [41], increasing the risk of small intestinal bacterial overgrowth (SIBO) [42] that is linked to DGBI [41, 43].

We observed prior NSAID use to be independently associated with a gut‐to‐brain pathway dominance in FD alone and GERD alone. NSAIDs may induce upper GI ulceration and increase intestinal permeability [44, 45, 46]. Injury identified at upper endoscopy, including ulceration, is associated with an increased risk of subsequent Parkinson's disease, supporting the concept NSAIDs may induce an important gut‐brain pathway disturbance [47]. We also found prior antibiotic use to be an independent predictor of a gut‐brain pathway in FD, IBS/FD overlap, IBS, and GERD, confirming previous findings [21]. Both NSAIDs and antibiotics can also alter the gut microbiota [46, 48]. In contrast, prior antidepressant use was found to be an independent predictor of having a brain –gut pathway in IBS, FD, overlap IBS/FD, and GERD. This is expected given antidepressants may have been used to treat the psychological disorder first.

Another factor that was an important predictor of a gut‐to‐brain pathway in the multivariate models for IBS, FD, IBS/FD overlap, and GERD, but not IBD, was a prior bout of gastroenteritis. This is in line with current research that suggests that transient or chronic gastrointestinal inflammation resulting from infectious gastroenteritis may play a major role in the initiation and pathogenesis of DGBI [49]. Numerous studies over the last three decades have shown that incident DGBI (both IBS and FD) may occur following acute gastroenteritis [49]. Of interest, some studies have shown that people with higher levels of background psychological distress are more likely to develop a post‐infectious IBS [50]. Having gastroenteritis at any time was associated with an increased probability of a brain‐to‐gut pathway first for IBS, FD, and overlap IBS/FD, although prior gastroenteritis was associated with a gut‐to‐brain pathway.

The lack of association of acute gastroenteritis with IBD is consistent with a recent meta‐analysis; although notably, there was very high heterogeneity, so pooling studies here was problematic [51].

In terms of those with evidence of a more dominant brain‐to‐gut pathway, the presence of alcohol‐related diagnoses was an independent risk factor for all DGBI and GERD. We hypothesize that alcohol‐related use disorders may be a surrogate for failure to adequately cope with psychological distress. *H. pylori* showed a small but independent association with a brain‐gut pathway in IBS, but this is potentially confounded by socioeconomic status, which we could not assess. Jones et al. observed a higher Townsend (socioeconomic disadvantage) score was an independent risk factor for an increased probability of a psychological disorder preceding a DGBI diagnosis [10]. Asthma was linked to a brain‐to‐gut pathway in IBS and FD, while eczema was a risk factor for a brain‐gut pathway in GERD. Both atopic and autoimmune diseases have been linked to IBS, GERD, and psychiatric disorders [22, 23].

We did not identify significant moderators of the order of IBD and psychological condition onset. While there was a comparatively smaller sample available for IBD, the sample size was not low in absolute terms. More likely, there is an innate difference between IBD and DGBI, and the mechanisms involved in the order of incidence for IBD appear to be quite different from those that we could study.

This study has several notable strengths. It used rigorous definitions of chronic GI conditions, including DGBI, GERD, and IBD based on standardized Read codes. The substantial follow‐up period for patients helped reduce the chance of misdiagnosis. Additionally, we deliberately excluded other organic conditions that could account for symptoms, improving the specificity of findings. The long naturalistic follow‐up and inclusion of subjects at the time of diagnosis provided objective data compared to retrospective recall typically used in other studies. The use of data from general practice patients increased the representativeness of the sample compared to hospital populations, which often include more severe cases. Pharmacological data from the THIN dataset has also been validated [25], adding reliability to medication‐related findings.

However, there are several study limitations to consider. The study was unable to directly assess DGBI diagnoses based on the Rome IV criteria, the current gold standard [52] but not commonly used in general practice [53]. Thus, the findings likely reflect real‐world data. Data on symptom onset were not available, which may affect interpretations of gut‐brain versus brain‐gut interactions, but this study reports on associations on the order of diagnosis rather than causality. On the other hand, a diagnosis implies a clinical assessment and appropriate diagnostic testing, which is arguably more robust than relying on only a symptom. In addition, while there may have been a lag between symptom onset and diagnosis, this lag likely occurs for both GI and psychological disorders and thus would not particularly bias the results in one direction. It could also be argued that diagnosis is more useful, as by the time patients have sought health care, their symptoms are usually more severe or limiting and are of a sufficient duration to warrant a diagnosis of a DGBI or psychological disorder rather than just a mild occurrence that may disappear on its own and not qualify as disease. The reasons for prescribing medications such as proton pump inhibitors (PPIs) or NSAIDs were unknown. It is possible that patients were prescribed PPIs to treat symptoms of FD before an official diagnosis was given, but in any case, this would indicate that the association between PPI use and a gut‐brain pathway in FD is under‐estimated. While patients' access to other healthcare providers was not captured, where they may have been given a formal diagnosis of a GI or psychological disorder, we suggest it would be unlikely such a diagnosis would not be recorded in primary care, and patients can't have more than one primary care provider in the UK. The choice of moderators for incidence order was limited by the dataset, and area under the curve (AUC) analyses suggest other unmeasured factors contribute to incidence order. Important variables such as socioeconomic status or environmental influences on the gut microbiome (e.g., diet, lifestyle, medication use, family history) could not be assessed.

Our findings may have important implications for clinical practice. Understanding that brain‐gut pathways are bidirectional in DGBI but one direction may be predominant, and determining which came first may provide insights into developing a more personalized and effective targeted treatment plan. For example, knowledge that factors such as prior PPI use, antibiotics, and gastroenteritis can signal that the condition is more likely gut‐brain (peripherally) driven theoretically may guide the clinician toward targeted treatment with a gut‐focused intervention such as dietary modifications or a probiotic, while if a brain‐to‐gut pathway is likely predominant it may be more useful to focus on adding psychological interventions. This approach needs formal testing in randomized trials.

In conclusion, we found support for prior medication usage and gastroenteritis playing an important role in generating gut‐to‐brain pathway disturbances first resulting in GI disease, and later resulting in psychological disturbances. The risk factor profile that has emerged potentially implicates changes in the intestinal microbiome as important but requires confirmation.

## Author Contributions

N.A.K. involved in study conception and design, data analysis and interpretation of results, and manuscript drafting and preparation. M.P.J. involved in study conception and design, acquisition of data, data analysis, and manuscript drafting. A.S. involved in interpretation of results and manuscript drafting. G.H. involved in interpretation of results and manuscript drafting. N.J.T. involved in study conception and design, acquisition of data, acquiring funding, interpretation of results, and manuscript drafting.

## Conflicts of Interest

The authors declare no conflicts of interest. Prof. Gerald Holtmann received unrestricted educational support from the Falk Foundation. Research support was provided via the Princess Alexandra Hospital, Brisbane by GI. Therapies Pty Ltd., Takeda Development Center Asia, Pty Ltd., Eli Lilly Australia Pty Ltd., F. Hoffmann‐La Roche Ltd., MedImmune Ltd., Celgene Pty Ltd., Celgene International II Sarl, Gilead Sciences Pty Ltd., Quintiles Pty Ltd., Vital Food Processors Ltd., Datapharm Australia Pty. Ltd. Commonwealth Laboratories, Pty Ltd., Prometheus Laboratories, FALK GmbH & Co KG, Nestle Pty Ltd., Mylan, and Allergan (prior to acquisition by AbbVie Inc.). Dr. Holtmann is also a patent holder for a biopsy device to take aseptic biopsies (US 20150320407 A1). Prof. N. Talley. Disclosure: GutSee Ltd. (consulting microbiome, 2025), Brown University, Agency for Health Care Research and Quality (fiber and laxation) (2024), Rome Foundation (member gastroduodenal committee) (present), Biocodex (FD diagnostic tool) (present), Microba (consulting microbiome, 2025), Comvita Manuka Honey (FD trial consulting) (2025), BluMaiden (microbiome 2025) outside the submitted work. In addition, Dr. Talley has a patent Nepean Dyspepsia Index (NDI) 1998, a patent Licensing Questionnaires Talley Bowel Disease Questionnaire licensed to Mayo/Talley, “Diagnostic marker for functional gastrointestinal disorders” Australian Provisional Patent Application 2021901692, “Methods and compositions for treating age‐related neurodegenerative disease associated with dysbiosis” US Application No. 63/537,725. Financial support: Dr. Talley is supported by funding from the National Health and Medical Research Council (NHMRC) to the Center for Research Excellence in Digestive Health, and he holds an NHMRC Investigator grant.

## Data Availability

Data were obtained from the THIN system under an ethics approval which prohibits sharing by the authors.
